# A New Metric for Quantifying the Relative Impact of Risk Factors on Loss of Working Life Illustrated in a Population of Working Dogs

**DOI:** 10.1371/journal.pone.0165414

**Published:** 2016-11-09

**Authors:** Geoffrey Caron-Lormier, Naomi D. Harvey, Gary C. W. England, Lucy Asher

**Affiliations:** 1 School of Veterinary Medicine and Science, University of Nottingham, Sutton Bonington Campus, Leicestershire, United Kingdom; 2 Centre for Behaviour and Evolution, Henry Wellcome Building, Newcastle University, Newcastle, United Kingdom; Shanxi University, CHINA

## Abstract

In a resource-limited world, organisations attempting to reduce the impact of health or behaviour issues need to choose carefully how to allocate resources for the highest overall impact. However, such choices may not always be obvious. Which has the biggest impact? A large change to a small number of individuals, or a small change to a large number of individuals? The challenge is identifying the issues that have the greatest impact on the population so potential interventions can be prioritised. We addressed this by developing a score to quantify the impact of health conditions and behaviour problems in a population of working guide dogs using data from Guide Dogs, UK. The cumulative incidence of different issues was combined with information about their impact, in terms of reduction in working life, to create a work score. The work score was created at population-level to illustrate issues with the greatest impact on the population and to understand contributions of breeds or crossbreeds to the workforce. An individual work deficit score was also created and means of this score used to illustrate the impact on working life within a subgroup of the population such as a breed, or crossbreed generation. The work deficit scores showed that those removed for behavioural issues had a greater impact on the overall workforce than those removed for health reasons. Additionally trends over time illustrated the positive influence of interventions Guide Dogs have made to improve their workforce. Information highlighted by these scores is pertinent to the effort of Guide Dogs to ensure partnerships are lasting. Recognising that the scores developed here could be transferable to a wide variety of contexts and species, most notably human work force decisions; we discuss possible uses and adaptations such as reduction in lifespan, quality of life and yield in production animals.

## Introduction

Epidemiological studies of a health or behavioural issues typically begin by investigating the prevalence, or incidence of the disorder of interest. Characterising the incidence or prevalence of a health or behaviour issue within and between populations allows identification of potential risk factors. It is also important to understand the impact of an issue on an affected population, be the population people or animals. The impact of interest may vary depending on the study, for example, the impact could be by reduction in lifespan [1]; reduction in yield (e.g. milk yield reduction in cattle, [2]); reduction in quality of life (e.g. in people, [3], or animals, [4]); treatment cost [5]; length of hospitalization [6]; or reduction in days worked [7]. Studies which focus on the impact outcome of an issue may be more informative about the impact of a given issue than prevalence studies, but impact outcomes alone do not allow decision makers to compare the impact of different conditions at population-level. In order to prioritise which issues to assign limited resources to, with regard to research, treatment or prevention, it is vital that the relative impact of different issues is quantified. Therefore, an important challenge is to find a relevant measure of the population-level impact of health or behavioural issues. We consider this challenge in relation to reduction in working life in a working guide dog population.

Studies identifying risk factors for increased incidence of health issues are common in humans and animals, with both genetic and environmental risk factors typically being identified. For example, in dogs both genetic [8], and environmental [9] risk factors have been identified for atopic dermatitis. Signalment (breed, age, sex) and other physical characteristics can be important risk factors for disease; in dogs this could include breed, age and sex (e.g. influencing the presence of cranial cruciate ligament rupture, [10] and number of limbs affected, [11]) or fluid characteristics such as obesity [12]). Undesirable behavioural issues appear to follow the same trend. For example, studies of separation-related disorders in dogs have shown it to be partly heritable [13], whilst also influenced by multiple environmental factors [14, 15] in addition to individual differences such as the personality of a dog and interactions between the dog and owner personality [16].

In our previous works Caron-Lormier and colleagues used a historic dataset to understand both the incidence and impact of health and behavioural issues in working guide dogs, in terms of reduced days of working life [17, 18]. These studies give a good description of the situation, integrating incidence and impact information to reveal which disorders had the most overall impact on the population. For instance, we reported on both the incidence and impact on working life of health issues found in working guide dogs and argued that musculoskeletal issues were the most important category of health related issues because of their high incidence [17]. This fits with data from pet dogs, which suggests musculoskeletal issues are commonly seen by veterinarians [19, 20]. In guide dogs the incidence of musculoskeletal issues also reflected the impact, resulting in the greatest reduction in working life compared with other health issues [17].

With regards to the effect of undesirable behaviour on withdrawal of working guide dogs, we reported that the issues affecting the greatest number of guide dog partnerships were environmental anxiety, training related breakdowns, and fear/aggression [18]. However, the issues that led to the greatest reduction in working life were fear/aggression, chasing, and attentiveness. In this case, the incidence was not an accurate reflection of the impact in terms of loss of working life.

In a resource-limited world, organisations have to choose carefully where to allocate resources for the highest overall impact. However, such choices may not always be obvious: which has the biggest impact? A large change to a small number of individuals, or a small change to a large number of individuals? The challenge, therefore, is to identify the health and behavioural issues that have the greatest impact on the overall population so that we may prioritise potential interventions. In previous studies of pedigree dog health, it was suggested to develop a Breed-Disorder Welfare Impact Scores (BDWIS) incorporating the severity, the prevalence, and the impact on animal welfare of the different detrimental issues [21, 22]. Ultimately, these metrics, or scores, aim to facilitate the decision-making process when action is needed on such issues.

In the context of working individuals (humans or animals), if the issues lead to a removal from service, the loss of working life is another relevant measure of impact on the population, here the work force. The European Study of the Epidemiology of Mental Disorders (ESEMeD) project investigated both quality of life, and work loss days over a one-month period in people suffering from various mental and physical health disorders. Quantification of these two variables allowed them to conclude that mental health disorders were more important than common physical disorders in determining both quality of life and working ability [7].

Guide Dogs UK is one of the largest working dog organizations in the world. They provide mobility to blind and partially sighted, in part by breeding, training and supporting dogs to perform the role of a mobility aid. Guide Dogs take measures to ensure partnerships between dogs and their owners are lasting. This enables partnerships between a dog and a Guide Dog Owner to last as long as possible but certain health and behaviour issues can reduce working life. Dogs may need to stop working because they are no longer performing their role adequately or because it is no longer good for their welfare to continue working. Whilst Guide Dogs will be taking steps to ensure partnerships are lasting at an individual-level they also need to understand how best to ensure more partnerships last longer at a population-level. To enable such decisions within Guide Dogs, or other working dog organisations, a quantitative metric is required that summarises such information to identify the reasons for removal from working service that have the greatest impact on the organisation [17, 18].

As an illustration, German Shepherd Dogs (GSDs) are a commonly used breed for police and military working dogs [23, 24] and they are also used in guiding the visually impaired. The average working life of GSDs within Guide Dogs (UK) was much below that of the other breeds [17, 18], so one could argue that GSDs should be removed from the Guide Dogs population and be replaced by more “successful” breeds. On the other hand, GSDs make up less than 5% of the working guide dog population so removing them would not have a big impact on the overall work force.

The aim of this study was to develop a metric combining information on incidence and impact (reduction in working life) of the different health and behavioural reasons for withdrawal commonly found in working guide dogs. To do so, we calculated these Work Scores at two different levels: the population-level (WSp) and the strata-level (WSs). We also discuss the use of these scores as a decision-making tool with potential impacts upon breeding and training priorities within Guide Dogs (UK), recognizing that such a score could be useful as a decision-making tool in other contexts, both in animals and in people.

## Material and Methods

### Guide Dogs and their data

Details on Guide Dogs (UK) were given in [17] and in [18]. Therefore, we only briefly describe it here. Guide Dogs (UK) is the current working name of the Guide Dogs for the Blind Association. It was founded in 1931 and is now the “world’s largest breeder and trainer of working dogs” [25]. Guide Dogs breed around 1,300 puppies every year, the majority of which will go through training and a process of selection, from which those who are suitable will be paired with a visually impaired person when they are approximately two years of age.

There are five stages in the training of a guide dog: breeding, puppy walking, early training, advanced training, and finally partnership training. Qualified dogs are then matched with a visually impaired person, and the relationship may last up to eight years. Most dogs (~70%) will reach retirement, whilst about 14%, and 16%, will be withdrawn for health, and behaviour, related issues, respectively [17, 18]. We define here Retirement as healthy end of service, typically when dogs have worked for about 8 years. In contrast, Withdrawn dogs are dogs that did qualify as working guide dogs and were subsequently withdrawn from service because of a health or behavioural issue that prevented them from continuing to work. The study was approved by Guide Dogs, in accordance with the University of Nottingham's institutional guidelines and received ethical approval from the School of Veterinary Medicine and Science ethics committee. All data on which the conclusions rely are presented in the main paper in the form of tables and figures.

### Classification of the health and behavioural withdrawal groups

We used the health and behavioural withdrawal groups as defined in [17] and [18]. The number of dogs (and the associated mean working life where possible) in the different groups of breed, overall withdrawal reasons, health withdrawal reasons, behavioural withdrawal reasons, as well as their associated combinations, are shown in the following Tables: 1, 2, 3 and 4.

**Table 1:** Table of the number of dogs in each breed and the associated mean working life (MWL). L, Labrador; GR, Golden retriever; GSD, German shepherd dog. Crossbreeds given as sire x dam. *indicates the dam was an F1 crossbreed.

Breed	Frequency	MWL	Proportion
L	3303	2707	36.7%
GRxL	2434	2712	27.1%
GR	1009	2645	11.2%
LxGR	833	2720	9.3%
Other	435	2601	4.8%
GSD	427	2216	4.7%
LxGR*	313	2526	3.5%
LxL*	131	2801	1.5%
GRxGR*	109	2745	1.2%

**Table 2:** Frequency and proportion of the different types of working outcomes and their associated mean working life (MWL).

Working outcome	Frequency	MWL	% Population
Retired	6465	3103	71.9%
Withdrawn-Behaviour	1310	1152	14.5%
Withdrawn-Health	1219	2001	13.5%

**Table 3:** The number of dogs in the health withdrawal groups and the associated mean working life (MWL). % Pop. is the proportion of dogs in each withdrawal group relative to the total population. % Withdrawn is relative to the withdrawn population.

Health withdrawal group	Freq	MWL	% Pop.	% Withdrawn
Retired	6465	3103	71.9%	NA
Musculoskeletal	385	2003	4.3%	17.4%
General health deterioration	174	2272	1.9%	7.8%
Nervous Sensory	169	1777	1.9%	7.6%
Cancer	141	2220	1.6%	6.4%
Skin Condition	88	1343	1.0%	4.0%
Eye	71	2139	0.8%	3.2%
Endocrine	43	2044	0.5%	1.9%
Gastrointestinal	38	1892	0.4%	1.7%
Non-Specific	36	2310	0.4%	1.6%
Cardiovascular	31	2134	0.3%	1.4%
Urogenital	18	1842	0.2%	0.8%
Immune	14	1928	0.2%	0.6%
Respiratory	11	1814	0.1%	0.5%

**Table 4:** Table of the number of dogs in the behavioural withdrawal groups and the associated mean working life (MWL). % Pop. is the proportion of dogs in each withdrawal group relative to the total population. % Withdrawn is relative to the withdrawn population.

Behaviour withdrawal group	Freq.	MWL	% Pop.	% Withdrawn
Retired	6465	3103	71.9%	NA
Environmental Anxiety	321	1207	3.6%	14.5%
Willingness/Confidence	311	1523	3.5%	14.0%
Fear/Aggression	226	818	2.5%	10.2%
Social Behaviour	144	1084	1.6%	6.5%
Chasing	128	943	1.4%	5.8%
Attentiveness	76	955	0.8%	3.4%
Distraction	63	1090	0.7%	2.8%
Excitability	36	1113	0.4%	1.6%
Body Sensitivity	5	949	0.1%	0.2%

### The Work Scores

A population-level metric was created, called a Work Score (WSp), which combines the proportion of dogs in a given group *g* with the proportion of reduction in working life (compared to the retired (i.e., Old) population). We note that the group *g* could be either a withdrawal group or a breed:

$$WSp_{g}=\frac{AffDogs_{g}}{AvailDogs}*\frac{WorkLife_{g}-RefWorkLife}{RefWorkLife}$$ (1)

Eq 1 can be split into two parts. First, we calculate the cumulative incidence (frequency over a given period of time, also known as incidence proportion) for each withdrawal group *g* (e.g., Musculoskeletal) by dividing the number of dogs in that group (*AffDogs*_*g*_) by the total number of dogs available (*AvailDogs*). Second, we calculate the change in working life relative to the retired group by calculating the difference between the mean working life in that group *g* (*WorkLife*_*g*_) and the retired population (*RefWorkLife*), and dividing this difference by the mean working life of the retired population (*RefWorkLife*). Finally, we multiply the two parts to get the work score at the population-level for group *g*.

Work scores were also calculated at the individual (dog) level (WSi), by removing part one of the equation (the reference to the number of dogs affected), leaving only the proportion of lost (or gained) working life (represented as a percentage) for a particular group *g*. The equation becomes:

$$WSi_{g}=\frac{WorkLife_{g}-RefWorkLife}{RefWorkLife}$$ (2)

Work scores can take any value between -100 and 100; negative values represent a negative impact on the strata at hand, values around 0 suggest no (or very little) impact, and positive values would imply a positive impact on the population from group *g*. A mean ‘Work Deficit Score’ can be calculated for each factor of interest (breed or generation for example). Work Deficit Scores for some groups may not represent the impact on the overall population if the group represents a small proportion of the population. In this example, instead of summing population impact, the Work Deficit Score is therefore illustrating the service longevity of the individual working dog, and the impact of the various withdrawal reasons on the guide dog owners in terms of loss of potential working life.

The work scores were developed to aid the identification of the withdrawal issues that have the greatest impact in terms of working length. The scores will therefore help the prioritisation of any intervention procedures in decision-making situations, but are not statistical tests. As such no statistical tests are associated with the work scores. All these scores are calculated relative to the reference group (the retired dogs) and it would be possible to use a different value, for instance a target reference working life.

Here, we consider the work scores for breeds, generation levels (F0 being pure breed, F1 first generation crossbreeds of two F0, and F1b backcrosses between an F1 and an F0), and the different withdrawal groups (health and behaviour related).

## Results

### Basic summary

The Labrador was the most common breed over the last 20 years comprising 37% of the working population, 10% higher than the Golden retriever x Labrador crossbreed (Table 1). Most dogs (71.9%) reached retirement without behavioural or health issues, whilst 14.5% (and 13.5%) were withdrawn for behavioural (and health) reasons (Table 2). The three main health withdrawal groups were Musculoskeletal (385 dogs), Nervous sensory (180 dogs), and General health deterioration (174 dogs), whilst the groups Respiratory, Immune, and Urogenital contained, on average, less than one dog per year (Table 3). The three main behavioural withdrawal groups were Environmental anxiety (321 dogs), Willingness/Confidence (311 dogs), and Fear/Aggression (226 dogs), whilst only five dogs were withdrawn for Body Sensitivity (Table 4).

### Population-level work scores (WSp)

**Fig 1:**
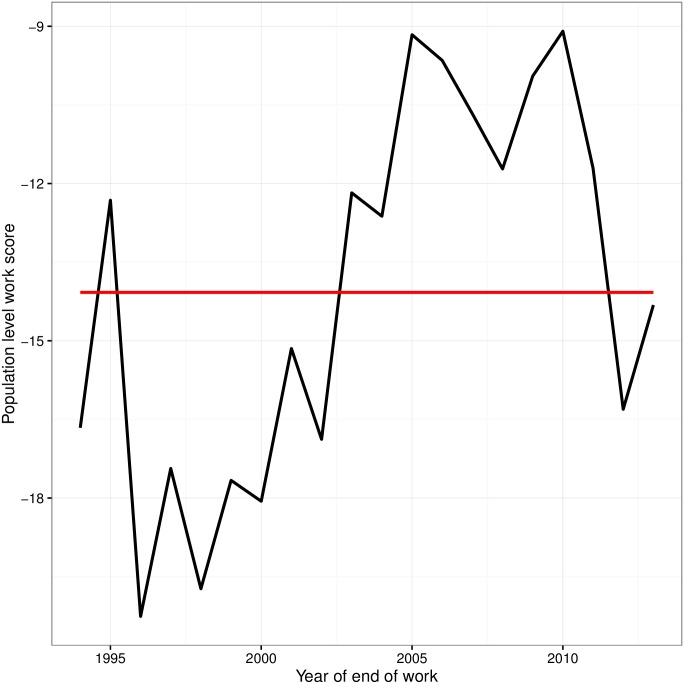
Combined population level work scores (WSp) over time.

**Fig 2:**
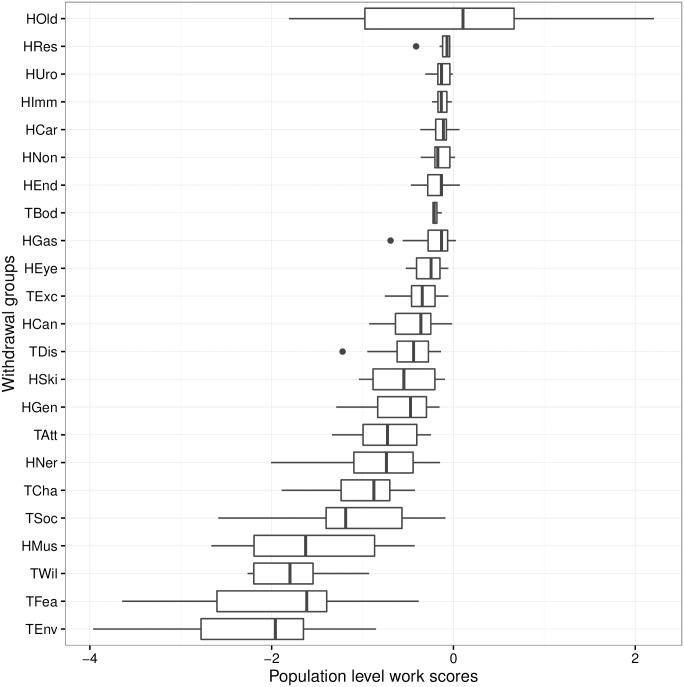
Boxplot of the work scores (WSp) over for each withdrawal group over 20 years. Groups preceded by a ‘H’ indicate health withdrawals (Old, retired; Can, cancer; Car, cardiovascular; Gas, gastrointestinal; Gen, general health deterioration; Mus, musculoskeletal; Ner, nervous/sensory; Non, nonspecific; Ski, skin), and groups preceded by a ‘T’ indicate behaviour withdrawals (Env, Environmental Anxiety; Soc, Social Behaviour; Exc, Excitability; Wil, Willingness/Confidence; Att, Attentiveness; Dis, Distraction; Fea, Fear/Aggression).

**Fig 3:**
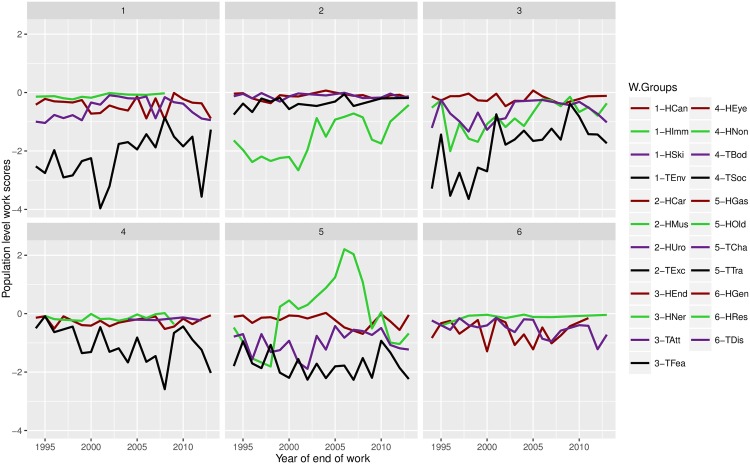
Change over time of the work scores (WSp) for each withdrawal group. The different groups were distributed into six different panels on the figure, and the groupings have no biological significance. Groups preceded by a ‘H’ indicate health withdrawals (Old, retired; Can, cancer; Car, cardiovascular; Gas, gastrointestinal; Gen, general health deterioration; Mus, musculoskeletal; Ner, nervous/sensory; Non, nonspecific; Ski, skin), and groups preceded by a ‘T’ indicate behaviour withdrawals (Env, Environmental Anxiety; Soc, Social Behaviour; Exc, Excitability; Wil, Willingness/Confidence; Att, Attentiveness; Dis, Distraction; Fea, Fear/Aggression).

The values for WSp changed over time, from -20% in 1996 up to -9% in 2005 and 2010, whilst the mean over the last 20 years is -14% (Fig 1). If all dogs reached retirement, the values of WSp would be 0.0. The withdrawal groups with the greatest impact on WSp over the last 20 years, were Environmental Anxiety, Fear/Aggression, and Willingness/Confidence, for the behaviour based groups, and Musculoskeletal for the health groups. All these groups have a population-level work score around -2% (Fig 2). Fig 3 displays the WSp values over time for each year of the 20-year period, for each of the withdrawal groups.

On pane one, we find that Environmental Anxiety had the strongest impact, with two troughs in 2001 and 2012, representing almost 4% loss of working life each time. On pane two, the health group Musculoskeletal has the most impact, but also shows an improvement over time from 2000 onwards, and getting close to 0 in the last few years. From pane three, the Fear/Aggression group had the most impact, particularly before 2000 with scores close to the -4% mark; its score seems to stabilise at just over the -2% mark in recent years. Pane four shows the withdrawal group Social Behaviour decreasing over time and reaching the -2% mark in the last few years. In pane five, we find that the Retired group varies from -2% to +2%, and the group Willingness/Confidence is stable around the -2% mark. Pane six, and last, shows the three groups General health deterioration (chronic non-specific generalised health-related debility causing reduced working ability), Respiratory, and Distraction, with around -1% work scores for the last 20 years.

**Fig 4:**
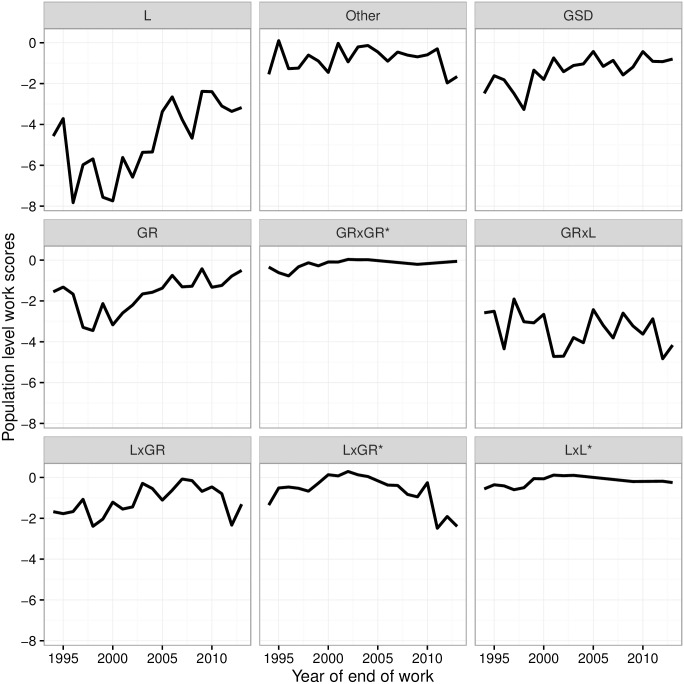
Population level work scores (WSp) over for each breed over time. L, Labrador; GR, Golden retriever; GSD, German shepherd dog. Crossbreeds given as sire x dam. *indicates the dam was a crossbreed.

The population-level work scores for the different breeds are shown in Fig 4. We find that the Labrador’s WSp increases over time from -8% to -3%. Labradors had the greatest impact on the work force pre-2000, with this impact shifting to Golden retriever x Labrador crossbreed more recently. The remaining breeds had, and still have, moderate impact on the total work force with work scores above -4% mark.

**Fig 5:**
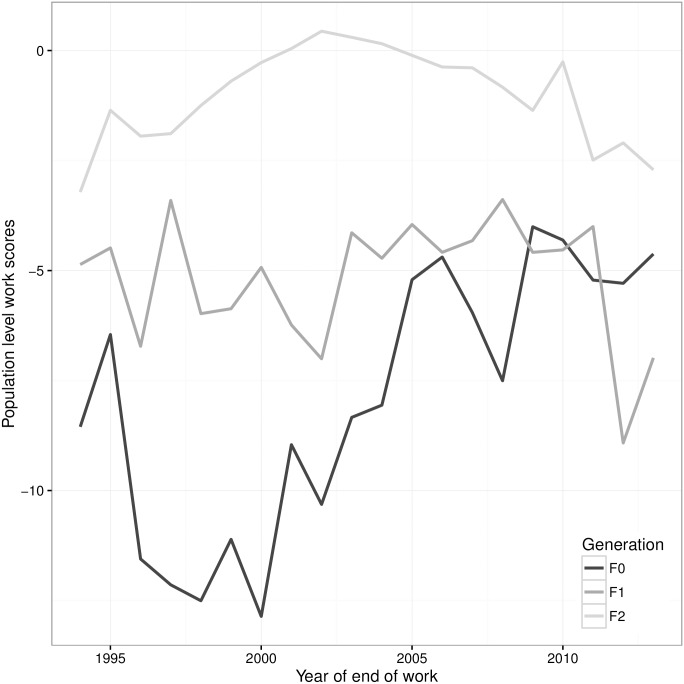
Population work scores (WSp) over time for purebred (F0), first generation (F1) and backcrossed (F1b) crossbreeds.

In order to investigate the impact of different generations of crossbreeds, breeds were grouped by generation of cross, from F0 (pure) to F1b (backcross). We find that the F0 generation had a strong impact (<-10%) around the year 2000, with its impact reducing to -5% more recently. The impact of F1 on WSp seems stable around -5% (apart from a short drop in 2012). F1b generations showed a reduction of impact, even reaching a positive impact in 2002, and then its impact declined towards -2.5% in the last few years (Fig 5).

#### Work deficit scores (WSi)

**Fig 6:**
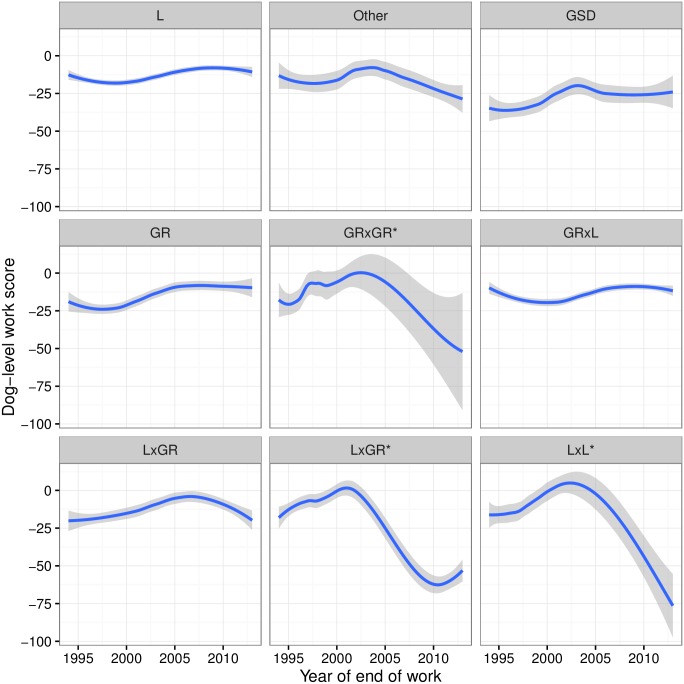
Dog-level work score (WSi) for each breed over time. L, Labrador; GR, Golden retriever; GSD, German shepherd dog. Crossbreeds given as sire x dam. *indicates the dam was a crossbreed.

The individual work deficit score WSi of the different breeds represent the mean performance loss or gain per individual compared to a gold standard comparator. In this case the mean performance was the working life for each dog as compared to the working life if reaching retirement (Fig 6).

We find that the breeds Labrador, Golden retriever, and Golden retriever x Labrador, seem stable at -12%. Conversely, the Labrador x Golden crossbreed showed an increase in work score (towards 0.0) until 2006, followed by a steady decline reaching -20% in 2013. German shepherd dogs have a mean loss of working life of 25% since 2005. The backcrosses of Golden and Labrador retrievers have seen a decline in their work scores since early 2000, now achieving, on average, between -50% and -75% less working life.

**Fig 7:**
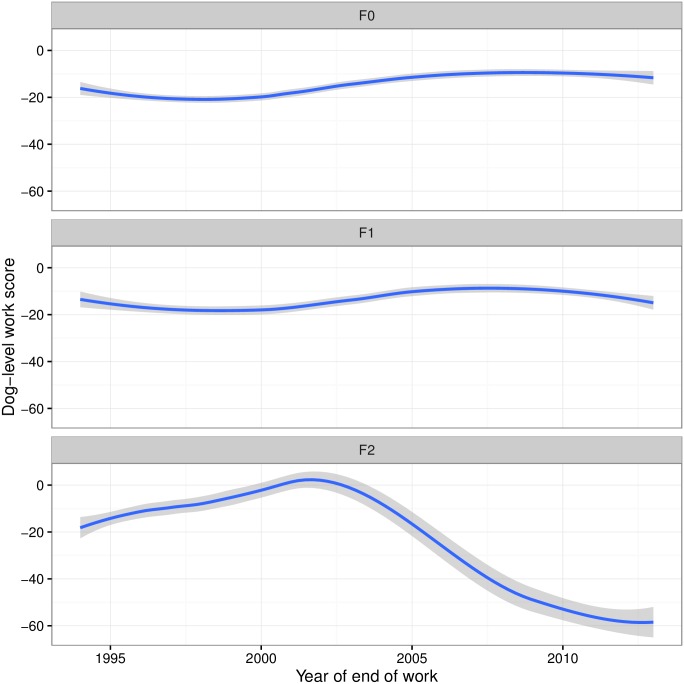
Dog-level work score (WSi) for purebred (F0), first (F1) and backcrossed (F1b) generation crossbreeds over time.

This trend is particularly noticeable when breeds are grouped by generation levels. We find that F0 and F1 generations are similar to each other with a mean of (individual) 10%-15% loss of working life. Conversely, F1b generation showed a decrease in WSi (or an increase in loss of working life) from 2002 onwards (Fig 7).

## Discussion

The aim of this work was to develop a tool to aid decision-making on resource allocation that integrated incidence and impact information. We developed a metric, which we called the work score, to identify the factors, such as breeds or withdrawal groups, which had the greatest impact on the working life of the Guide Dogs workforce at population level and attempted to understand the impact at strata-level. These metrics should help Guide Dogs to prioritise interventions that will lead to an improvement, not only, of the overall working life of the entire workforce, but also, of the individual quality of working guide dogs. This work on guide dogs can be seen as both informative for those with interest in working dogs and as illustrative of the potential of work score to applications in other areas. For example, the work score could be easily transferred to understand the reasons for retirement in humans and the impact on the overall workforce. Different outcomes (other than retirement) could be used such as a reduction in yield in other contexts. This work also illustrates the inherent power of using historic datasets from working dog organisations since datasets with a 20-year span are usually rare or not easily accessible. It is worth noting that this metric was calculated after dogs had been grouped by “end of work year”. Dogs could be easily grouped by other dates, such as qualification or birth, to produce a similar metric. There is an inherent limitation in that dogs can work up to eight years and therefore dogs ending their service in the same year could have gone through different training systems.

## Population-Level Work Scores

Population-level work scores were calculated for the different withdrawal groups and breeds. These scores were relative to the retired group so that any negative scores would represent a negative impact in the form of a loss of working life. We found that the withdrawal groups with the highest impact on the work force were Environmental anxiety, Fear/Aggression, and Willingness/Confidence. The only health withdrawal group with a mean work score of less than -1% was Musculoskeletal. These results make biological sense as health issues tend to occur when dogs are getting older whereas behavioural issues arise younger dogs, excepting ‘Willingness/Confidence’ issues which can occur at any age [18]. From these results, we would suggest that interventions leading to an increase in the work scores for these four withdrawal groups would have the greatest impact on the work force. Increasing the work scores could be achieved, at least in principle, by either, decreasing the number of dogs affected by these withdrawal groups, or/and, increasing the working life associated with these withdrawal groups.

These population work scores can be followed over time to check whether there are noticeable trends, for example, in response to breeding selection or training and handling interventions for the behavioural groups. When looking at Musculoskeletal withdrawal reasons, we see that the group shows a definite upward trend since the early 2000s. This suggests that health issues falling under the Musculoskeletal group are having less of an impact, at the population level, in terms of working life. Such a change could be due to the introduction of hip-dysplasia screening in the early 2000s for the breeding stock. In the last year of the data, the work score associated with the Musculoskeletal group was higher than -0.5% which is a three-fold improvement from -2% pre-2000. Similarly, the Environmental Anxiety group seems to be improving over time, excepting the years 2001 and 2012; in the last year of the data the work score was just under -1% (compared to nearly -3% in 1997), which could be due to improvements in early life socialisation.

A work score of 0.0 would mean that, on average, a particular withdrawal group would have no better or worse impact than the reference “Retired”. Interestingly, the Retired group (HOld in the figures) has a variable work score over time. Since the mean working life (over the last 20 years) was used to calculate the work scores, some variation between years is perfectly sensible. It does illustrate that, considering that retired dogs comprise the majority of the population, increasing the working life associated with the retired group, even marginally, would have the largest impact with regards to increasing the overall work force performance. Interestingly there was a peak in HOld work scores around the year 2006, indicating that dogs were being retired later this year and improving the work-force impact. Whilst we don’t know what may have caused this in this case, such peaks and troughs could be used by organisations to trace back to changes in practice that had positive or negative impacts on the measurement outcome.

Looking at the breed work scores over time, we see that Labrador retrievers have improved by more than 50%, with a marked improvement from 2000 onwards. Golden retrievers seem to be following a similar trend. Such illustration of breed impacts could be very useful for identifying the breeds that have the highest impact on the work force at any given time.

### Individual-level work scores

Individual-level work scores can be considered surrogates for performance of a particular group (e.g. a given breed). They represent the potential impact on the guide dog owners in terms of how long the partnership lasted, and thus relevant to customers. This metric represents a loss of working life relative to the Retired group and allows us to see quickly how much working life is lost, on average for each group, for example each breed category. It is particularly useful when we follow these over time as we can highlight any trends.

The three breeds Labrador, Golden retriever, and Golden x Labrador crossbreed seem to be performing similarly, with work scores oscillating around the -10% mark, suggesting that on average a guide dog owner with a dog of one of these three breeds should not experience a loss of working life greater than 300 days (or 0.8 of a year). German shepherd dogs have on average a working life reduced by 25%, which seems stable since 2005. The most pronounced loss of working life is shown in the F1 backcrosses (GRxGR*, LxGR*, and LxL*) with a loss of working life of at least 50%. Whilst the same proportion of F1b dogs fail to reach retirement as other breeds, those that are withdrawn they appear to be more often withdrawn for those withdrawal groups that lead to the greatest loss of working life; so they are withdrawn earlier. Removing these backcrosses from the work force, and replacing them with a combination of the three “top” performing breeds would be expected to improve the total workforce of the population. Replacing F1b dogs would also improve the individual experience of guide dog owners that would have otherwise received one of these backcrosses, as their dogs would likely work for longer, although it would have little impact on the overall population performance as these three backcrosses had small population-level work scores.

### Application to other systems

The work scores developed here focused on dogs performing their function as mobility aids and thus the impact outcome considered was length of working life compared to dogs retired for old age. This could be seen as directly comparable to working life in people where different reasons for retirement could be considered in comparison to reaching the prescribed retirement age. For instance, in the context of work and pension, one could list all the reasons for early retirement (positive i.e. early retirement due to financial stability, or negative i.e. ill health or work stress) and the associated number of people leaving work for these reasons and the associated mean loss of working life. This would easily create a work score that could help identify the reasons with the greatest impact on the working human population. If considering negative reasons for stopping work, much like this example in dogs, the overall impact on the workforce could be considered for particular reasons to target interventions to help more of the workforce reach retirement age. Similarly, reasons for leaving a workforce, or company, could be considered to prioritise resources to intervene to retain the greatest number of staff. For example different potential interventions have been identified for retaining experienced nurses in a health care system [26]. If the reasons for nurses leaving were documented, the population work score could be used to target these interventions to the group that could most improve the time length nurses were retained. The strata work scores could reveal which group were leaving nursing earliest. Similarly, different health reasons for exit or retirement from a workforce could be calculated e.g. using data like that collected by the European Study of the Epidemiology of Mental Disorders (ESEMeD) project [7]. For this study the impact focused upon reduction in days worked, but the score could be adapted to consider other outcomes in people or animals for example: length of life, length of hospitalisation or treatment, or, theoretically, reduction in Quality of Life (QoL). Using reduction in QoL relative to a relevant reference group or baseline scores would be a useful marker of the impact on the individuals affected. However, existing tools that measure QoL often fail to clearly define it, and few are rigorously validated or appropriate for use with multiple issues [4] so this may not be possible for the present. For production animals calculating work scores could be simpler as the work score could be translated to days of production lost or a reduction in yield. An innovative study using a dynamic model to understand the timing of culling decisions that were either maximised according to the economic interest of the farmer or the welfare interest if the cows illustrates that in stratified populations, such as a dairy cowherd, different subgroups in the population may be contributing differentially to the overall yield [27]. A work score approach could be used in such stratified populations as a basic metric for understanding the contributions of the different groups of the population or the impacts of the different diseases (or other production limiting factors) to consider which interventions would have the most impact at a population-level.

## Conclusions

Previous studies have described and analysed the odds of dogs being withdrawn for the different withdrawal reasons and their associated reduction in working life [16, 17]. The current study goes further by creating a metric (the work score) that allows for the identification of the factors that have the highest impact on the work force at the population-level, and at the individual level for different groups. Such metrics could have great potential within working dogs population as decision-making tools for prioritising intervention strategies to improve service quality. Furthermore, the population-level work score can be used to monitor population-level trends in working longevity in response to changes in training, work selection, or breeding practices. Overall, the work score can be used to help understand different levels of impact from the various factors commonly found in any working dog organisation. The metric described here is also transferable to workforce decision-making in people, and could be adapted to consider impacts of interest in other species including production animals.

## Abbreviations

WSp: A population-level metric was created, which combines the proportion of individuals in a given group *g*, with the proportion of reduction in a measure of impact (in this case length of working life) as compared to a reference population
WSi: the proportion of lost (or gained) measure of impact (in this case length of working life) for a particular group *g*
F0: pure breed dog
F1: first generation crossbreed
F1b: F1 backcross
L: Labrador
GR: Golden retriever
GSD: German shepherd dog

## References

[1] Pierce JP, Stefanick ML, Flatt SW, Natarajan L, Sternfeld B, Madlensky L, et al: Greater survival after breast cancer in physically active women with high vegetable-fruit intake regardless of obesity. J Clin Oncol 2007, 25:2345–2351. 10.1200/JCO.2006.08.6819 17557947 PMC2274898

[2] Green LE, Hedges VJ, Schukken YH, Blowey RW, Packington AJ: The impact of clinical lameness on the milk yield of dairy cows. J Dairy Sci 2002, 85:2250–2256. 10.3168/jds.S0022-0302(02)74304-X 12362457

[3] Devinsky O, Westbrook L, Cramer J, Glassman M, Perrine K, Camfield C: Risk Factors for Poor Health-Related Quality of Life in Adolescents with Epilepsy. Epilepsia 1999, 40:1715–1720. 10612334 10.1111/j.1528-1157.1999.tb01588.x

[4] Belshaw Z, Asher L, Harvey ND, Dean RS: Quality of life assessment in domestic dogs: an evidence-based rapid review. Vet J 2015, 206:203–212. 10.1016/j.tvjl.2015.07.016 26358965 PMC4641869

[5] Simon GE, VonKorff M, Barlow W: Health care costs of primary care patients with recognized depression. Arch Gen Psychiatry 1995, 52:850–856. 7575105 10.1001/archpsyc.1995.03950220060012

[6] Macht M, King CJ, Wimbish T, Clark BJ, Benson AB, Burnham EL, et al: Post-extubation dysphagia is associated with longer hospitalization in survivors of critical illness with neurologic impairment. Crit Care 2013, 17:R119 10.1186/cc12791 23786755 PMC4057203

[7] Alonso J, Angermeyer MC, Bernert S, Bruffaerts R, Brugha TS, Bryson H, et al: Disability and quality of life impact of mental disorders in Europe: results from the European Study of the Epidemiology of Mental Disorders (ESEMeD) project. Acta Psychiatr Scand 2004, 109:38–46. 15128386 10.1111/j.1600-0047.2004.00329.x

[8] Sousa CA, Marsella R: The ACVD task force on canine atopic dermatitis (II): genetic factors. Vet Immunol Immunopathol 2001, 81:153–157. 11553376 10.1016/s0165-2427(01)00297-5

[9] Hill PB, DeBoer DJ: The ACVD task force on canine atopic dermatitis (IV): environmental allergens. Vet Immunol Immunopathol 2001, 81:169–186. 11553378 10.1016/s0165-2427(01)00298-7

[10] Whitehair JG, Vasseur PB, Willits NH: Epidemiology of cranial cruciate ligament rupture in dogs. J Am Vet Med Assoc 1993, 203:1016–1019. 8226247

[11] Grierson J, Asher L, Grainger K: An investigation into risk factors for bilateral canine cruciate ligament rupture. Vet Comp Orthop Traumatol 2011, 24:192–196. 10.3415/VCOT-10-03-0030 21327290

[12] German AJ: The Growing Problem of Obesity in Dogs and Cats. J Nutr 2006, 136:1940s–1946s 10.1093/jn/136.7.1940S 16772464

[13] King JN, Simpson BS, Overall KL, Appleby D, Pageat P, Ross C, Chaurand JP, et al: Treatment of separation anxiety in dogs with clomipramine: results from a prospective, randomized, double-blind, placebo-controlled, parallel-group, multicenter clinical trial. Appl Anim Behav Sci 2000, 67:255–275. 10760607 10.1016/s0168-1591(99)00127-6

[14] Flannigan G, Dodman NH: Risk factors and behaviors associated with separation anxiety in dogs. J Am Vet Med Assoc 2001, 219:460–466. 11518171 10.2460/javma.2001.219.460

[15] Takeuchi Y, Ogata N, Houpt KA, Scarlett JM: Differences in background and outcome of three behavior problems of dogs. Appl Anim Behav Sci 2001, 70:297–308. 11179553 10.1016/s0168-1591(00)00156-8

[16] Konok V, Kosztolányi A, Rainer W, Mutschler B, Halsband U, Miklósi Á: Influence of owners’ attachment style and personality on their dogs’(Canis familiaris) separation-related disorder. PLoS One 2015, 10:e0118375 10.1371/journal.pone.0118375 25706147 PMC4338184

[17] Caron-Lormier G, England GCW, Green MJ, Asher L: Using the incidence and impact of health conditions in guide dogs to investigate healthy ageing in working dogs. Vet J 2016, 207:124–130. 10.1016/j.tvjl.2015.10.046 26616425

[18] Caron-Lormier G, Harvey ND, England GCW, Asher L: Using the incidence and impact of behavioural conditions in guide dogs to investigate undesirable behaviours in working dogs. Sci Reports 2016, 4 14;6:23860. 10.1038/srep23860 PMC4831008 27075868

[19] Nielsen TD, Dean RS, Robinson NJ, Massey A, Brennan ML: Survey of the UK veterinary profession: common species and conditions nominated by veterinarians in practice. Vet Rec 2014:vetrec–2013. 10.1136/vr.101745 PMC3995283 24570401

[20] Robinson NJ, Dean RS, Cobb M, Brennan ML: Paper: Investigating common clinical presentations in first opinion small animal consultations using direct observation. Vet Rec 2015, 176:463 10.1136/vr.102751 25564472 PMC4431344

[21] Collins LM, Asher L, Summers JF, Diesel G, McGreevy PD: Welfare epidemiology as a tool to assess the welfare impact of inherited defects on the pedigree dog population. Anim Welf 2010, 19:67–75.

[22] Collins LM, Asher L, Summers J, McGreevy P: Getting priorities straight: Risk assessment and decision-making in the improvement of inherited disorders in pedigree dogs. Vet J 2011, 189:147–154. 10.1016/j.tvjl.2011.06.012 21742521

[23] Wilsson E, Sinn DL: Are there differences between behavioral measurement methods? A comparison of the predictive validity of two ratings methods in a working dog program. Appl Anim Behav Sci 2012, 141:158–172.

[24] Arvelius P, Strandberg E, Fikse WF: The Swedish Armed Forces temperament test gives information on genetic differences among dogs. J Vet Behav 2014, 9:281–289.

[25] Guide Dogs: Guide Dogs {UK} Charity for the Blind and Partially Sighted (Date of access: 01/02/2016). 2015.

[26] Cohen JD: The aging nursing workforce: How to retain experienced nurses. J Healthc Manag 2006, 51:233 16916117

[27] Langford FM, Stott AW: Culled early or culled late: economic decisions and risks to welfare in dairy cows. Anim Welf 2012, 21(Supplement 1):41–55.

